# Supplementary material for: Iran’s great scientific divergence: The Middle-Income trap and the Political Economy of Science Policy
Source: PLoS One. 2026 Jun 3;21(6):e0349342. doi: 10.1371/journal.pone.0349342 (PMC13232799; doi:10.1371/journal.pone.0349342)
Supplement: Attachment — Submitted filename: Reviewers_Plos_One.pdf [file pone.0349342.s003.pdf]

Reviewers Plos One

Ehsan Roohi

April 2026

Editorial Letter and Comments

PLOS ONE
Apr 15, 2026, 10:41 PM
to Ehsan
PONE-D-26-00074
Iran's Great Scientific Divergence: The Middle-Income Trap and the Political Economy of Science Policy
PLOS One

Dear Dr. Roohi,

Thank you for submitting your manuscript to PLOS ONE. After careful consideration, we feel that it has merit but does not fully meet PLOS ONE's publication criteria as it currently stands. Therefore, we invite you to submit a revised version of the manuscript that addresses the points raised during the review process.

==============================

The analysis prioritizes sensitivity to the study's ambitious scope (scientometrics + political economy) while highlighting critical gaps that undermine its rigor and interpretive validity.

1. Overreliance on a single donor country (South Korea): The manuscript states the SCM algorithm assigned a weight of 1.0 to South Korea as the sole donor for Synthetic Iran. SCM's strength lies in weighted combinations of multiple donor countries to approximate the treated unit; a single donor eliminates the method's core advantage (counterfactual validity via multivariate matching) and reduces it to a simple country comparison. No justification is provided for why the donor pool was limited (or why no other countries (e.g., Taiwan, Turkey) contributed to the synthetic control), nor is a placebo test (a required robustness check for SCM) reported to validate the causal impact of the 1979 Revolution.

   Response: We thank the reviewer for this important methodological comment. We agree that the earlier formulation, in which South Korea received a weight of 1.0, did not adequately leverage the core logic of SCM.

In the revised manuscript, we have therefore re-estimated the SCM using a broader multilateral donor pool and report the resulting donor weights explicitly. In the revised baseline specification, the synthetic control is no longer a single-country comparison. Instead, it is constructed from a weighted combination of donor countries, with China receiving the dominant weight and smaller positive contributions from Israel, Greece, and Singapore.

We emphasize that this result should be interpreted as an optimization outcome of the SCM procedure rather than as a claim that China is the single most historically analogous case to Iran. SCM does not select donors based on contemporary geopolitical status or narrative similarity alone; rather, it selects the convex combination of donor units that minimizes pre-treatment mismatch in the joint space of observed predictors and lagged outcome values. In our data, China provides the strongest numerical contribution to reproducing Iran's pre-1979 structural profile once GDP per capita, tertiary enrollment, government expenditure, trade openness, and the pre-treatment publication path are considered together. The smaller weights on Israel, Greece, and Singapore further reduce the remaining imbalance.

To address the reviewer's concern regarding robustness, we have also added in-space placebo tests and RMSPE-based comparisons to assess whether Iran's post-1979 divergence is unusually large relative to placebo reassignments within the donor pool. These results show that Iran's post-treatment gap lies in the upper tail of placebo deviations, supporting the interpretation of a substantial structural break rather than a trivial artifact of fit. In addition, we now report specification sensitivity checks using alternative predictor sets. Across these variants, the qualitative conclusion remains unchanged: Iran's post-1979 scientific trajectory falls substantially below its comparison-based counterfactual.

Finally, to avoid conflating different counterfactual logics, we now distinguish clearly between the revised SCM benchmark and the separate scaled-Korea benchmark. The former is selected by pre-treatment fit, whereas the latter is retained only as a historically motivated growth-proxy comparison. We believe these revisions directly address the reviewer's concern about the earlier overreliance on a single donor country.

2. The SCM uses four World Bank predictors (GDP per capita, tertiary enrollment, government expenditure, trade openness) but provides no rationale for excluding other critical covariates of scientific output (e.g., R&D spending as a % of GDP, number of researchers per capita, international scientific collaboration rates). Omission of these variables risks omitted variable bias in the synthetic control.

   Response: We thank the reviewer for this important point. We agree that R&D expenditure, researcher intensity, and international collaboration are conceptually relevant covariates for scientific output and impact.

In revising the manuscript, we therefore clarified both the rationale for our baseline predictor set and the limits imposed by historical data coverage.

Our baseline SCM specification retains four structural covariates from the World Bank, GDP per capita, tertiary enrollment, government expenditure, and trade openness, because these variables offer the broadest and most usable historical coverage for the pre-treatment period relevant to our SCM design. Since the treatment year is 1979, the SCM must reproduce Iran's pre-1979 trajectory using predictors that are available with sufficient consistency across the donor pool before treatment. In contrast, variables such as R&D expenditure as a share of GDP and researchers per capita are only sparsely available for most countries in the pre-1979 period, and therefore could not be incorporated into the baseline SCM without substantially shrinking the donor pool or introducing severe missing-data imbalance.

We also note that international scientific collaboration rates differ conceptually from the macro-structural predictors used in the baseline SCM. Collaboration intensity is itself partly an outcome of scientific integration and institutional openness, and for the historical period considered here it is more naturally treated as a bibliometric robustness variable than as a core World Bank predictor. For this reason, we did not include collaboration share in the baseline SCM optimization.

To address the reviewer's concern more directly, we revised the manuscript in three ways. First, we clarified in the Data and Methods section that the baseline SCM uses four World Bank predictors because they provide the most reliable historical coverage for pre-1979 matching across the donor pool. Second, in the Limitations of the Study subsection, we now state explicitly that the SCM benchmark remains constrained by predictor availability and donor-pool composition, and therefore should be interpreted alongside placebo tests, predictor-specification checks, and alternative benchmarks rather than as a single definitive counterfactual. Third, we expanded the robustness analysis through a broader donor pool and alternative predictor specifications, showing that the qualitative conclusion remains unchanged even when the comparison set is widened.

Accordingly, our revision does not claim that the four baseline predictors are the only conceptually relevant determinants of scientific output. Rather, they are the most appropriate predictors for a historically balanced pre-treatment SCM implementation in this case. We have revised the text to make this rationale explicit and to distinguish clearly between conceptually desirable covariates and those that are empirically usable in a long-horizon SCM design.

Changes made in the manuscript: We revised the *Data and Methods* section to clarify the rationale for the baseline SCM predictor set, expanded the discussion in the *Limitations of the Study* subsection regarding predictor availability and donor-pool sensitivity, and added additional

robustness analyses in the SCM results section.

3. The model assumes Iran would have sustained South Korea's post-1979 growth rates while starting from a larger 1978 publication base. This ignores structural differences between pre-1979 Iran and South Korea: Iran's pre-revolution scientific growth was fueled by oil rent-funded state spending(not industrial demand), while South Korea's growth was tied to export-led industrialization. The model does not account for these structural drivers, making its projection of Iran "surpassing South Korea" by 2024 empirically ungrounded. - No sensitivity analysis: No alternative growth proxies (e.g., Taiwan's trajectory, a weighted average of Asian Tigers) are tested to triangulate the "knowledge deficit" estimate. The manuscript relies heavily on Model D for its core quantitative claim but does not assess how sensitive the 551,000 publication deficit is to alternative counterfactual specifications.

4. The manuscript attributes Iran's sub-1.0 FWCI (below global average) exclusively to domestic policy and rentier state logic but understates Scopus indexing biases for non-Western/isolated countries: Iranian research is often published in regional (non-Scopus) journals, and international collaboration (a key driver of FWCI) is constrained by geopolitical sanctions (a factor acknowledged but not empirically quantified). No adjustment is made for these biases, leading to an overattribution of the quality gap to domestic policy.

   Response: We thank the reviewer for this important and constructive comment. We agree that the earlier version of the manuscript placed too much interpretive weight on domestic policy and rentier-state logic when discussing Iran's citation-impact gap. In the revised manuscript, we have substantially reframed this section to make clear that the observed FWCI shortfall should not be interpreted as a pure or bias-free measure of intrinsic national research quality.

   Specifically, we made four substantive revisions.

   First, we revised the discussion of the "quantity–quality paradox" to emphasize that the observed citation-impact gap reflects multiple interacting mechanisms, operating at both the domestic and international levels, rather than a single causal pathway. The text now explicitly states that the FWCI shortfall is interpreted as a joint outcome of domestic incentive structures, reduced international collaboration opportunities, and bibliometric visibility constraints.

   Second, we strengthened the manuscript's treatment of international collaboration by adding new empirical evidence from SciVal for 1996–2024. We inserted a new cross-country figure showing international collaboration share by year for Iran and comparator systems, and a second figure showing Iran's FWCI and international collaboration share over time. These additions were made to address the reviewer's concern that sanctions and

reduced integration into international networks were acknowledged but not empirically illustrated. The new figures show that Iran's internationally co-authored publication share has generally remained below that of the most globally integrated comparator systems for much of the period, supporting a more cautious interpretation of the observed FWCI gap.

Third, we revised the relevant interpretation in the Results section to clarify that citation-based indicators derived from Scopus reflect the indexed publication universe and may underrepresent research published in regional or non-Scopus-indexed outlets. We now state explicitly that the observed FWCI gap should not be read as a pure measure of intrinsic research quality alone.

Fourth, we expanded the Limitations section by adding a new paragraph on uneven bibliometric systems, sanctions, international collaboration constraints, and possible non-Scopus undercoverage. The revised text now explains that for countries facing geopolitical isolation and weaker integration into high-visibility collaboration networks, observed citation impact may reflect not only domestic incentive structures, but also differential visibility, collaboration opportunity, and index coverage. Accordingly, the manuscript now treats FWCI as an observed indexed-impact measure, rather than as a complete or bias-free indicator of national scientific quality.

These revisions do not eliminate the importance of domestic policy and incentive structures, but they do materially rebalance the interpretation. The revised manuscript therefore no longer attributes the quality gap exclusively to internal policy logic; instead, it presents the gap as the product of both institutional incentives and international bibliometric/integration constraints

5. The manuscript reports Iran's 12% share of top 10% cited papers (2024) but does not field-normalize this metric by discipline. Iran's research output is concentrated in specific fields (e.g., engineering, basic science); a failure to field-normalize means the "top 10%" comparison to countries with more diversified research portfolios (e.g., Netherlands, Singapore) is apples-to-oranges.

   Response: In response to the reviewer's concern, we conducted an additional field-specific robustness analysis using SciVal FWCI data for six major subject areas: Engineering, Medicine, Physics and Astronomy, Computer Science, Materials Science, and Mathematics. Because FWCI is field-normalized by design, this analysis provides a discipline-sensitive comparison that reduces the risk of conflating portfolio composition with research impact. We added a new figure in the revised manuscript summarizing average field-specific FWCI over 2020–2024 and show that, although Iran is relatively closer to some comparator systems in selected fields, substantial gaps remain relative to the highest-performing systems. We therefore revised the manuscript to clarify that the top-10% indica-

tor is treated as supplementary evidence, while the main interpretation of cross-country quality differences relies more heavily on FWCI and the new field-specific robustness check.

6. In 2.1 Contradictory Publication Volume Data - The abstract and counterfactual analysis state a cumulative knowledge deficit of 551,000 publications by 2024 (SCM model), but Section 4.10 reports Iran's actual cumulative output* as ˜864,675 papers and Synthetic Iran's as ˜1.42 million (a gap of 555,325—a ˜4,000 paper discrepancy). No explanation is provided for this numerical inconsistency, which erodes confidence in the core quantitative finding.

   Response: We thank the reviewer for identifying this issue. We agree that the earlier version of the manuscript gave the impression that a single cumulative publication-deficit figure could be treated as the paper's core quantitative finding, while different sections reported closely related but not identical rounded values. In the revised manuscript, we have addressed this problem by reframing the counterfactual analysis more explicitly as a benchmark-sensitive exercise rather than a single-number estimation exercise.

   Specifically, we now distinguish clearly between annual output gaps and cumulative shortfalls, and we state explicitly that cumulative shortfall estimates are benchmark-specific and may vary modestly across model summaries depending on donor composition, terminal-year convention, and rounding. We have therefore removed reliance on one headline cumulative-deficit number in the abstract and conclusion, and instead emphasize that the qualitative finding of a durable post-1979 divergence is robust across specifications, whereas the precise quantitative magnitude remains sensitive to benchmark construction.

   These revisions were made to eliminate the appearance of numerical inconsistency and to align the presentation of the results with the paper's actual methodological position.

7. - Section 4.1 claims Iran published 518 papers in 1978, while South Korea published 260. However, the Crossref data in Figure 6 (1960–1995) shows Iran's 1978 output as ˜400 papers (not 518). No reconciliation is provided for this Scopus vs. Crossref data mismatch.

   Response: We thank the reviewer for pointing this out. We agree that, in the previous presentation, the manuscript did not make sufficiently explicit that the pre-1979 annual count reported in Section 4.1 and the historical reconstruction shown in the Crossref-based figure come from two different data series with different purposes.

   In the revised manuscript, we clarify this distinction explicitly. The 1978 value of 518 refers to the main Scopus-indexed publication series used for the paper's primary longitudinal comparisons. By contrast, the Crossref-based pre-1996 figures are introduced as historical reconstructions in-

tended to recover broad trend patterns during the period of incomplete Scopus coverage. They are therefore approximate and are not expected to reproduce the exact annual levels of the Scopus series one-to-one.

To avoid confusion, we have revised the text in Section 4.1 to state that the 1978 figure refers specifically to the Scopus-indexed series, and we have added a clarifying sentence in the Crossref reconstruction section explaining that the Crossref-based figures are meant for historical trend approximation rather than exact numerical equivalence with the main Scopus counts. This change resolves the apparent mismatch and makes the role of the two datasets more transparent.

8. The manuscript states Iran's university teaching staff fell from **16,877 (1979–80) to 9,042 (1982–83) (a loss of 7,835) but cites only a single source (Shahrzad Mojab, 1991) for this critical statistic. No cross-verification with Iranian government archives, UNESCO data, or other secondary sources is provided, and no breakdown (e.g., full-time vs. part-time staff) is given to contextualize the loss of research-active faculty (the key driver of scientific output).

   Response: We thank the reviewer for this important comment. We agree that the university staffing contraction is a substantively important part of the historical argument and therefore requires clearer documentation and contextualization.

   In response, we revisited the source base for this statistic. The figures originally cited from Mojab (1991) are not isolated to a single secondary source. We identified additional independent reproductions of the same Ministry of Culture and Higher Education statistical series, including sources that report the same total teaching-staff counts for 1979–80 (16,877) and 1982–83 (9,042), as well as a gender breakdown of these totals. Specifically, the reproduced series reports a decline from 2,455 to 1,424 in female teaching staff and from 14,422 to 7,618 in male teaching staff over the same interval. We have therefore revised the manuscript to cite these corroborating sources alongside Mojab.

   At the same time, we agree with the reviewer that the available historical statistics refer to aggregate university teaching staff and do not cleanly separate research-active full-time faculty from part-time or sessional personnel. We therefore clarified the text so that this staffing contraction is interpreted as evidence of a major shock to university teaching and research capacity, rather than as a precise census of active researchers alone.

   These revisions strengthen the evidentiary basis of the claim while also making its interpretive limits more transparent.

9. - The claim that pre-1979 Iran's scientific output "surpassed South Korea and Taiwan" is qualified only by 1978 publication counts; no per-capita normalization is provided for this comparison (Iran's population

in 1978 was ˜36 million, South Korea's ˜37 million, Taiwan's ˜18 million—Taiwan's per-capita output was far higher). This omission misrepresents Iran's pre-revolution scientific standing.

Response: We thank the reviewer for this valuable comment. We agree that the earlier wording in the manuscript could be read too strongly if interpreted as a population-normalized comparison. In the revised manuscript, we therefore clarified that the late-1970s comparison refers to absolute Scopus-indexed publication counts rather than per-capita scientific intensity.

To examine the point directly, we conducted a simple 1978 per-capita check using the Scopus-indexed publication counts and approximate contemporary population totals. This exercise shows that Iran remained ahead of South Korea in publications per million people, but substantially below Taiwan on that metric. Specifically, the resulting 1978 publications-per-million values are approximately 13.9 for Iran, 7.1 for South Korea, 30.8 for Taiwan, and 10.6 for Turkey. We therefore revised the text so that it no longer implies an unqualified pre-1979 superiority over Taiwan.

The manuscript now states more precisely that Iran had become regionally competitive in scientific scale and growth momentum prior to 1979, while also acknowledging that Taiwan exhibited substantially stronger per-capita publication intensity. We also added a linking sentence in the per-capita subsection to make this qualification explicit in the normalized analysis.

10. - The manuscript labels 1980–1999 Iran's "lost decade" (Section 4.2) but later refers to it as a "lost decade" (singular) in Section 1, creating a terminological inconsistency (20 years vs. 10 years). The 1990s are framed as part of the "stagnation" period, but Scopus data shows Iran's publication output surpassed its 1978 peak in 1995—a critical inflection point that is not adequately analyzed or contextualized.

    Response: We thank the reviewer for this careful observation. We agree that the earlier wording created a terminological inconsistency by referring to the broader 1980–1999 period as a "lost decade," even though that interval spans two decades and includes more than one internal phase.

    In the revised manuscript, we corrected this issue by distinguishing more clearly between the sharp collapse of the 1980s and the slower recovery dynamics of the 1990s. We no longer use "lost decade" as a label for the full 1980–1999 interval. Instead, the revised text characterizes 1980 through the late 1990s as a prolonged post-revolutionary stagnation phase, composed of (i) an initial collapse phase in the 1980s and (ii) a slow and incomplete recovery phase in the 1990s.

    We also revised Section 4.2 to address the reviewer's second point directly. The manuscript now states explicitly that the mid-1990s crossing of Iran's 1978 publication level is a meaningful inflection point. However, we interpret this threshold crossing not as full recovery, but as the end of the

immediate trough and the start of a transition phase. The stronger acceleration that becomes visible after 2000 is therefore distinguished from the mid-1990s threshold recovery.

These revisions make the periodization more precise and better aligned with the empirical pattern in the publication data.

11. - The manuscript contains duplicate content in Sections 5 and 7 (both titled Mechanisms of Divergence: A Comparative Policy Analysis), with identical analysis of South Korea's Technology Development Promotion Law (1972) and Iran's Cultural Revolution. This redundancy is a major editorial flaw and disrupts the narrative flow.

    Response: We thank the reviewer for pointing this out. We agree that the earlier draft contained an editorial redundancy in which the comparative policy-analysis discussion was repeated, disrupting the narrative flow. In the revised manuscript, this duplication has been removed. The discussion of South Korea's Technology Development Promotion Law (1972), Iran's Cultural Revolution, and the comparative institutional interpretation now appears only once, in a single consolidated section titled "Mechanisms of Divergence: A Comparative Policy Analysis." This revision eliminates the redundancy and improves the continuity of the manuscript's argument.

12. - Section 6 (Policy Implications) is followed by a near-identical Section 8 (Policy Implications), with the same three policy pivots (volume-to-impact, diaspora integration, breaking the rentier link) restated verbatim. No new analysis or refinement is added in Section 8, making it redundant.

    Response: We thank the reviewer for noting this editorial redundancy. We agree that an earlier draft contained a duplicated policy-implications section. In the revised manuscript, this duplication has been removed. The policy discussion now appears only once, in a single consolidated section titled "Policy Implications: Escaping the Trap," where the three policy pivots are presented in their final form. This revision eliminates the redundancy and improves the manuscript's narrative flow.

13. The manuscript applies the MIT framework to Iran's scientific system but fails to operationalize the MIT for scientometrics. The MIT is an economic concept (factor accumulation vs. productivity-driven growth); the manuscript does not define how this translates to a scientific MIT (beyond the "quantity-quality paradox") or test it against alternative theories (e.g., national innovation systems theory). No empirical test (e.g., regression analysis of publication volume vs. FWCI) is provided to confirm Iran is "trapped" in the scientific accumulation phase.

    Response: We thank the reviewer for this important conceptual comment. We agree that the earlier version of the manuscript invoked the Middle-Income Trap (MIT) framework more as an interpretive analogy than as an explicitly operationalized scientometric concept.

In response, we revised the manuscript in three ways. First, we now define a "scientific MIT" more explicitly as a pattern in which a research system succeeds in expanding publication scale but shows persistently weaker conversion of that scale into normalized scientific influence. In this formulation, the relevant empirical symptom is a decoupling between output accumulation and impact upgrading, rather than low output alone.

Second, we clarified the relationship between the MIT framing and alternative theoretical perspectives, especially national innovation systems theory. In the revised text, the MIT framework is presented not as a substitute for national innovation systems theory, but as a more specific lens for interpreting one developmental pattern within it: prolonged accumulation without commensurate transition to a high-impact innovation regime.

Third, we added a simple empirical check using Iran's SciVal-period data (1996–2024). This analysis shows that annual publication output increased from 877 to 76,939 papers (about 87.7 times), while FWCI increased from 0.53 to 1.12 (about 2.11 times). Trend fits show very strong growth in log output over time ($R^2 \approx 0.914$), positive but more limited improvement in FWCI ($R^2 \approx 0.872$), and only partial conversion of scale growth into normalized citation influence in the FWCI–log(output) relationship ($R^2 \approx 0.723$). We therefore treat the MIT language as a pattern-based interpretation supported by the observed decoupling between scale growth and impact conversion, rather than as a claim of a formally identified economic trap in the strict original macroeconomic sense.

14. The manuscript attributes Iran's science policy failures to rentier state dynamics but understates post-2000 changes in Iran's oil economy (e.g., sanctions-induced decline in oil revenues, diversification efforts). No analysis is provided for how falling oil rents (2010s–2020s) impacted science funding—an important test of the rentier state thesis.

    Response: We thank the reviewer for this important point. We agree that the earlier version of the manuscript stated the rentier-state interpretation too statically and did not sufficiently acknowledge that Iran's oil economy changed substantially after 2000, especially during the sanctions-intensive and fiscally constrained period of the 2010s and 2020s.

    In response, we revised the manuscript in three ways. First, we now clarify in the theoretical section that the rentier-state argument is not meant to imply a temporally uniform post-2000 oil regime or a simple linear relationship between oil income and science funding. Instead, the argument is that a rent-dependent institutional structure can continue to shape the volatility, allocation logic, and weak industrial anchoring of science policy even when oil rents themselves become more unstable or constrained.

    Second, we added a qualification in the mechanisms section stating explicitly that the later post-2000 period was marked by sanctions-related

revenue compression, fiscal instability, and diversification pressures. We therefore interpret the rentier mechanism in this later period less as a story of abundant oil rents directly financing science, and more as a broader institutional pattern in which scientific funding remained vulnerable to externally driven fiscal cycles and reprioritization.

Third, we added a limitation clarifying that the manuscript does not assemble a direct annual series linking oil-rent fluctuations to science-budget allocations in the 2010s and 2020s. For this reason, the rentier-state interpretation is presented as an institutional-political explanation of the broader pattern rather than as a direct econometric demonstration of year-to-year funding effects.

These revisions make the argument more historically precise and better aligned with the reviewer's concern.

15. The manuscript frames China's scientific recovery (post-Cultural Revolution) as a "near-total reset followed by rapid growth" but ignores key differences (e.g., China's integration into the global economy post-1978, massive state R&D spending, lack of international sanctions). The China-Iran comparison is superficial and not theoretically justified.

    Response: We thank the reviewer for this important comment. We agree that the earlier wording on China risked sounding too schematic and did not sufficiently emphasize the major structural differences between China's post-1978 recovery environment and Iran's post-revolutionary trajectory.

    In response, we revised the manuscript to clarify that China is not treated as a theoretically equivalent recovery model for Iran. The revised text now states explicitly that China's post-Cultural-Revolution scientific expansion occurred under structural conditions that sharply distinguish it from Iran's later experience, including reintegration into the global economy, very large-scale state investment in science and technology, expanding industrial export capacity, and the absence of prolonged international sanctions comparable to those faced by Iran.

    We also clarified the role that China plays in the manuscript more generally. Where China appears in the comparative narrative, it is now framed as a contrast case of recovery after political disruption under much more favorable conditions of global integration and state capacity. Where China appears in the SCM results, we explicitly state that its donor weight is an optimization outcome of the synthetic-control procedure rather than evidence of deep theoretical equivalence with Iran.

    These revisions narrow the China-Iran comparison appropriately and make the manuscript's comparative logic more historically and theoretically precise.

16. The manuscript claims Iran's quantity-quality paradox is driven "not merely by external sanctions or war" but by domestic policy (bureaucratic incentives). However, it fails to empirically disentangle domestic

vs. external factors: no regression, difference-in-differences, or moderation analysis is provided to quantify the relative impact of sanctions (e.g., U.S. sanctions on scientific collaboration, export controls on lab equipment) vs. domestic incentive structures on FWCI and publication quality. This leads to an overemphasis on domestic policy and an underappreciation of geopolitical constraints.

Response: We thank the reviewer for this important comment. We agree that the revised manuscript should not be read as having causally disentangled the relative effects of domestic incentive structures and external geopolitical constraints on Iran's post-2000 citation-impact gap.

In response, we revised the text in two ways. First, we softened the interpretation of domestic policy incentives so that they are presented as one plausible contributing mechanism rather than as a uniquely identified driver of the quantity–quality paradox. Second, we added an explicit limitation stating that the manuscript does not implement a causal identification strategy – such as a regression-based decomposition, moderation design, or quasi-experimental framework – that would separately estimate the effects of sanctions, export restrictions, collaboration barriers, and domestic promotion incentives on FWCI or related quality indicators.

The revised manuscript therefore treats the quantity–quality paradox as the product of interacting domestic and external mechanisms. Our contribution is to document the pattern, illustrate key dimensions of international integration and visibility using collaboration evidence, and offer an institutionally grounded interpretation, while acknowledging that the relative causal weights of these mechanisms remain for future work.

17. The manuscript references Figure 1 (Scientific Middle-Income Trap) but then labels Iran's publication output as Figure 2, South Korea as Figure 3, etc.—but the text often mislabels figures (e.g., Section 4.5 refers to "Figure 8" for Iran's post-2000 output, but Figure 8 is the Crossref Top-10% metric for 1960–1995).

    Response: We thank the reviewer for identifying the figure-reference inconsistencies in the earlier version of the manuscript. We agree that the previous draft mixed manually written figure numbers with automatically renumbered figures after the insertion of new material, which created several incorrect references.

    In the revised manuscript, we corrected the figure citations systematically by replacing manual numeric references with label-based cross-references throughout the text. As a result, the Crossref reconstruction, the SciVal output/FWCI/top-10% discussion, the logarithmic trajectory comparison, the per-capita analysis, and the growth-momentum analysis are now all referenced using LaTeX labels rather than hard-coded figure numbers.

    We also corrected a formatting problem in the comparative policy table, where one row had become misaligned during revision. These changes

remove the figure-number inconsistencies and improve the internal consistency of the manuscript.

18. Table 1 (Comparative Scientometric Profile): The table reports Taiwan's total output as ˜600k (1996–2024) but South Korea's as ˜1.2M—but Scopus data for Taiwan (2000–2024) shows ˜50k–60k annual publications, meaning Taiwan's cumulative output (1996–2024) is ˜1.2M (not 600k). This is a critical data entry error that misrepresents Taiwan's scientific output.

    Response: We thank the reviewer for identifying the inconsistency in Table 1. We rechecked the underlying 1996–2024 output export used in the manuscript and found that the previously reported rounded totals in Table 1 were not fully consistent with the source file. In the revised manuscript, we corrected the total-output entries for all countries in Table 1 using the same underlying dataset. The corrected totals are: Iran = 980,305; South Korea = 1,736,423; Taiwan = 935,554; Singapore = 472,133; Netherlands = 1,443,404; and Israel = 549,328. This correction removes the numerical inconsistency and aligns the table with the longitudinal output series used elsewhere in the manuscript.

19. There is missing Citations in manuscript: Key claims (e.g., "Iran's research is concentrated in lower-tier venues") are not cited, and some references are duplicated (e.g., [3] and [13] are both Alice Amsden's *Asia's Next Giant*).

    Response: We thank the reviewer for this helpful comment. We agree that the manuscript should avoid unsupported interpretive claims and should use a clean, non-duplicative bibliography.

    In response, we made three changes. First, we reviewed the discussion sections and added citations to support claims that previously appeared without explicit reference support, especially those concerning the role of international collaboration in citation visibility and the use of publication-based incentives in Iran's academic evaluation system. Second, we removed duplicate bibliographic entries, including the duplicated record for Alice Amsden's *Asia's Next Giant*, which now appears only once in the reference list. Third, we corrected inconsistent citation keys in the manuscript so that all in-text references now match the bibliography file.

    These revisions improve both the evidentiary support and the internal consistency of the manuscript.

20. There is ambiguous Definitions in manuscript, for example

    - "Knowledge Deficit": The manuscript uses this term interchangeably to refer to annual publication gaps (Section 4.10) and cumulative publication gaps (abstract, conclusion) without a clear definition. This ambiguity confuses the core quantitative finding.

- “Export Discipline”: The term is defined for South Korea but not operationalized for Iran—no explanation is provided for how “export discipline” would translate to Iran’s economy (a rentier state with a small non-oil export sector) or what a “disciplined” Iranian science policy would look like in practice.

Response: We thank the reviewer for this important clarification request. We agree that the earlier version of the manuscript left two key terms insufficiently defined: “knowledge deficit” and “export discipline.”

First, we revised the manuscript to define these terms more explicitly. The text now distinguishes systematically between the *annual gap* (the year-specific difference between Iran’s observed output and a benchmark) and the *cumulative shortfall*. We further clarify that the term “knowledge deficit” is used only as shorthand for the *cumulative* publication shortfall over a specified period and should not be read interchangeably with the annual gap.

Second, we clarified the meaning of “export discipline” in the comparative discussion. In the revised manuscript, this term is no longer treated as a Korea-specific slogan but as a broader policy mechanism in which science and technology investment is disciplined by external performance criteria such as industrial upgrading, competitiveness, technological absorption, and demand from productive sectors. We also state explicitly that an analogous mechanism in Iran would not imply replication of South Korea’s export structure, but rather stronger non-rentier performance feedback linking science policy to productive-sector outcomes instead of publication counts alone.

These revisions make the manuscript’s conceptual language more precise and reduce the ambiguity identified by the reviewer.

21. - The manuscript concludes that the 1979 Revolution’s “generational cost” is due to lost institutional continuity but overgeneralizes this finding to all “political disruptions.” No analysis is provided for how other political disruptions (e.g., post-Soviet collapse) differ from Iran’s case (e.g., Iran’s retention of a centralized state vs. the Soviet Union’s dissolution), making the conclusion overly broad.

    Response: We thank the reviewer for this important clarification. In the revised manuscript, we now state explicitly that the paper does not generalize Iran’s experience to all political disruptions. We clarify that the post-Soviet case is used only as a broad contrast in recovery dynamics, not as a directly comparable institutional analogue, since it involved state dissolution and systemic fragmentation, whereas post-revolutionary Iran retained a centralized national state but underwent an internally driven ideological rupture. We also revised the conclusion to emphasize that Iran represents a historically specific configuration of disruption rather than a universal model of political shock.

22. The policy implications (e.g., "create islands of excellence") are prescriptive but uncontextualized: no analysis is provided for how these policies would be implemented in Iran's political system (e.g., Supreme Council of the Cultural Revolution's control over universities) or how to overcome bureaucratic resistance to reform.

    Response: We appreciate this criticism and agree with the reviewer. In the original submission, the policy section identified plausible reform directions but did not sufficiently discuss the institutional conditions under which such reforms could realistically be implemented in Iran. We have therefore revised the policy discussion to make it more explicitly conditional, politically situated, and implementation-oriented.

    The revised text now explains that reforms concerning faculty evaluation, research autonomy, diaspora engagement, and funding governance must be understood in relation to Iran's centralized higher-education structure, including the role of the MSRT, the PBO, and the Supreme Council of the Cultural Revolution. We also clarify that "islands of excellence" are not presented as a simple prescriptive formula, but as a constrained pilot-based strategy that might be feasible even in a highly centralized system through semi-protected institutes, specialized laboratories, and competitively funded research centers.

    In addition, the manuscript now explicitly acknowledges bureaucratic resistance as an endogenous feature of the existing system rather than an external implementation detail. Because publication-centered evaluation and centralized allocation mechanisms create institutional constituencies, reform cannot be assumed to occur frictionlessly. The revised text therefore emphasizes sequencing, demonstration effects, and gradual institutional differentiation as more realistic pathways than immediate system-wide transformation.

Minor Errors & Omissions

- Typos/Grammar: Frequent typos (e.g., "post2000" instead of "post-2000," "salami slicing" without definition, "Enqelab-e Farhangi" misspelled in one instance) and grammatical errors (e.g., run-on sentences, inconsistent verb tense) are present throughout the manuscript.

Response: We appreciate the reviewer's close reading. The manuscript has been carefully proofread and revised for typographical, grammatical, and stylistic consistency. Specifically, we standardized the hyphenation of compound expressions (e.g., "post-2000"), regularized the transliteration of Persian terms (including "Enqelab-e Farhangi"), defined previously informal or potentially ambiguous expressions such as "salami slicing," and revised several overly long sentences to improve readability. We also corrected minor punctuation and citation-formatting inconsistencies across the text. These revisions do not alter the substance of the argument, but they improve clarity, consistency, and presentation throughout the manuscript.

Major revisions are required to address these issues before the manuscript is suitable for publication in PLOS ONE.
. Be sure to:

1. Indicate which changes you require for acceptance versus which changes you recommend

2. Address any conflicts between the reviews so that it's clear which advice the authors should follow

3. Provide specific feedback from your evaluation of the manuscript

Please ensure that your decision is justified on PLOS ONE's publication criteria and not, for example, on novelty or perceived impact.
For Lab, Study and Registered Report Protocols: These article types are not expected to include results but may include pilot data.
==============================

Please submit your revised manuscript by end of May. If you will need more time than this to complete your revisions, please reply to this message or contact the journal office at plosone@plos.org. When you're ready to submit your revision, log on to https://www.editorialmanager.com/pone/ and select the 'Submissions Needing Revision' folder to locate your manuscript file.

Please include the following items when submitting your revised manuscript:

1. A letter that responds to each point raised by the academic editor and reviewer(s). You should upload this letter as a separate file labeled 'Response to Reviewers'.

2. A marked-up copy of your manuscript that highlights changes made to the original version. You should upload this as a separate file labeled 'Revised Manuscript with Track Changes'.

3. An unmarked version of your revised paper without tracked changes. You should upload this as a separate file labeled 'Manuscript'.

If you would like to make changes to your financial disclosure, please include your updated statement in your cover letter. Guidelines for resubmitting your figure files are available below the reviewer comments at the end of this letter.
If applicable, we recommend that you deposit your laboratory protocols in protocols.io to enhance the reproducibility of your results. Protocols.io assigns your protocol its own identifier (DOI) so that it can be cited independently in the future. For instructions see: https://journals.plos.org/plosone/s/submission-guidelines#loc-laboratory-protocols. Additionally, PLOS ONE offers an option for publishing peer-reviewed Lab Protocol articles, which describe protocols hosted on protocols.io. Read more information on sharing protocols at https://plos.org/protocols?utm_medium=editorial-email&utm_source=authorletters&utm_campaign=protocol

As the corresponding author, your ORCID iD is verified in the submission system and will appear in the published article. PLOS supports the use of ORCID, and we encourage all coauthors to register for an ORCID iD and use it as well. Please encourage your coauthors to verify their ORCID iD within the submission system before final acceptance, as unverified ORCID iDs will not appear in the published article. Only the individual author can complete the verification step; PLOS staff cannot verify ORCID iDs on behalf of authors.
We look forward to receiving your revised manuscript.

Kind regards,
Mehdi Borhani
Academic Editor
PLOS One

Journal Requirements

When submitting your revision, we need you to address these additional requirements.

1. Please ensure that your manuscript meets PLOS ONE's style requirements, including those for file naming. The PLOS ONE style templates can be found at

   - https://journals.plos.org/plosone/s/file?id=wjVg/PLOSOne_formatting_sample_main_body.pdf
   - https://journals.plos.org/plosone/s/file?id=ba62/PLOSOne_formatting_sample_title_authors_affiliat

2. Please note that your Data Availability Statement is currently missing [the repository name and/or the DOI/accession number of each dataset OR a direct link to access each database]. If your manuscript is accepted for publication, you will be asked to provide these details on a very short timeline. We therefore suggest that you provide this information now, though we will not hold up the peer review process if you are unable.

3. Please update your submission to use the PLOS LaTeX template. The template and more information on our requirements for LaTeX submissions can be found at http://journals.plos.org/plosone/s/latex.

4. Please ensure that you refer to Figure13 & 16 in your text as, if accepted, production will need this reference to link the reader to the figure.

5. We note you have included a table to which you do not refer in the text of your manuscript. Please ensure that you refer to Table 2 in your text; if accepted, production will need this reference to link the reader to the Table.

6. If the reviewer comments include a recommendation to cite specific previously published works, please review and evaluate these publications to determine whether they are relevant and should be cited. There is no requirement to cite these works unless the editor has indicated otherwise.

Response: We thank the Academic Editor for these final editorial instructions. We addressed each point as follows:

- The manuscript has been reformatted to comply with PLOS ONE's LaTeX and style requirements.
- The Data Availability Statement has been updated to include the required repository/access details.
- We checked all figure and table cross-references and ensured that Figure 13, Figure 16, and Table 2 are explicitly referred to in the main text.
- We also performed a final language and presentation review to remove minor inconsistencies and improve clarity in wording and formatting.

These revisions improve the manuscript's compliance with journal requirements and facilitate production without changing the substance of the study.
